# Involvement of the PD-1/PD-L1 Co-Inhibitory Pathway in the Pathogenesis of the Inflammatory Stage of Early-Onset Preeclampsia

**DOI:** 10.3390/ijms20030583

**Published:** 2019-01-29

**Authors:** Matyas Meggyes, Eva Miko, Adrienn Lajko, Beata Csiszar, Barbara Sandor, Peter Matrai, Peter Tamas, Laszlo Szereday

**Affiliations:** 1 Department of Medical Microbiology and Immunology, Medical School, University of Pecs, 12 Szigeti Street, 7624 Pecs, Hungary; meggyes.matyas@pte.hu (M.M.); miko.eva@pte.hu (E.M.); lajko.adri@gmail.com (A.L.); 2 Janos Szentagothai Research Centre, 20 Ifjusag Street, 7624 Pecs, Hungary; 3 1st Department of Internal Medicine, Medical School, University of Pecs, 13 Ifjusag Street, 7624 Pecs, Hungary; csiszar.beata@pte.hu (B.C.); sandor.barbara@pte.hu (B.S.); 4 Institute of Bioanalysis, Medical School, University of Pecs, 12 Szigeti Street, 7624 Pecs, Hungary; matraip@gamma.ttk.pte.hu; 5 Department of Obstetrics and Gynaecology, Medical School, University of Pecs, 17 Edesanyak street, 7624 Pecs, Hungary; tamas.peter@pte.hu

**Keywords:** preeclampsia, early-onset, PD-1, PD-L1, inflammation, immune checkpoint

## Abstract

The programmed cell death protein 1 (PD-1) receptor has been reported to downregulate T cell activation effectively via binding to its ligands PD-L1 or PD-L2 in a negative co-stimulatory manner. Little is known about the involvement of PD-1 mediated immunoregulation in pregnancy and in pregnancy-related disorders. In this work, we investigated the possible role of the PD-1 co-stimulatory pathway in the pathogenesis of the clinical phase of early-onset preeclampsia characterized by a systemic maternal inflammatory response. We performed a cross-sectional study for comparative analysis of phenotypic and functional characteristics of peripheral blood mononuclear cells in women with early-onset preeclampsia and third-trimester healthy pregnant controls. According to our findings, enhanced expression of either PD-1 or its ligand PD-L1, or both, on the cell surface of effector cells (T cells, natural killer (NK) cells, natural killer T (NKT)-like cells) and Tregs could be observed, but PD-1 expression did not correlate with effector cells exhaustion. These results suggest the failure of the axis to downregulate Th1 responses, contributing thereby to the exaggerated immunoactivation observed in early-onset preeclampsia.

## 1. Introduction

From an immunological point of view, pregnancy is considered to represent a permanent challenge for the maternal immune system, maintaining a delicate balance between protective immunity and tolerance [1]. Thereby, a major role was referred to immunoregulatory mechanisms overwriting ongoing immunoactivation both on a cellular (e.g., regulatory T cells (Treg)) and on a molecular (e.g., inhibitory receptors) level.

The actual hypothesis of successful T cell activation suggests the involvement of multiple incoming signals beside T-cell receptor (TCR) dependent recognition of the Major histocompatibility complex (MHC)-antigen complex [2,3,4]. Some of these signals are delivered by immune checkpoint molecules with negative regulatory potential. The programmed death-1 (PD-1) receptor is a 55 kDa transmembrane protein belonging to the Ig superfamily [5,6]. It has been reported to downregulate T cell activation effectively via binding to its ligands PD-L1 or PD-L2 in a negative co-stimulatory manner [6]. The PD-1 pathway is thought to be associated with the promotion of peripheral tolerance and subsequent prevention of immune-mediated tissue damage by favoring Th2 development and expansion [7,8]. Upon activation, PD-1 is widely expressed by different lymphocyte subsets (T cells, B cells, NK cells, NKT-like cells, and Mucosal associated invariant T (MAIT) cells) and by antigen presenting cells as well [9,10]. Its ligands, PD-L1 and PD-L2, are broadly distributed on both hemopoietic and non-hemopoietic cells, such as the decidua and the placenta [11,12,13].

Preeclampsia is a severe and common obstetrical disorder characterized by high blood pressure and proteinuria, combined with other systemic disorders. The clinical phase usually develops after the 20th week of gestation and affects approximately 7% of all human pregnancies [14]. Early-onset preeclampsia is defined as preeclampsia that develops before 34 weeks of gestation and is thought to be an implantation disorder with insufficient placentation in the pre-clinical phase [15]. Symptoms develop as a result of oxidative stress due to placental decompensation when fetal growth accelerates [16]. This clinical phase is characterized by a systemic maternal inflammatory response with the involvement of both innate and adaptive immunoactivation [17,18,19,20,21,22]. Because of the etiological role of the placenta, the only available definite treatment of controlling maternal symptoms is the initiation of delivery and complete removal of the placenta [14,16].

Little is known about the involvement of PD-1 mediated immunoregulation in pregnancy and in pregnancy-related disorders. In this study, we investigated the possible role of the PD-1 co-stimulatory pathway in the pathogenesis of the clinical phase of early-onset preeclampsia analyzing phenotypic and functional characteristics of peripheral blood lymphocytes.

## 2. Results

### 2.1. The Ratio of Different Lymphocyte Subpopulations in the Peripheral Blood of 3rd-Trimester Healthy Pregnant Women and in Women with Early-Onset Preeclampsia

We determined the ratio of different lymphocyte subpopulations in the peripheral blood of healthy pregnant and early-onset preeclamptic women. No differences were observed in the ratios of innate and adaptive effector lymphocytes (Table 1). 

### 2.2. PD-1 Expression by Different Lymphocyte Subsets of the Innate and the Adaptive Immunity of 3rd-Trimester Healthy Pregnant Women and in Women with Early-Onset Preeclampsia

**Figure 1:**
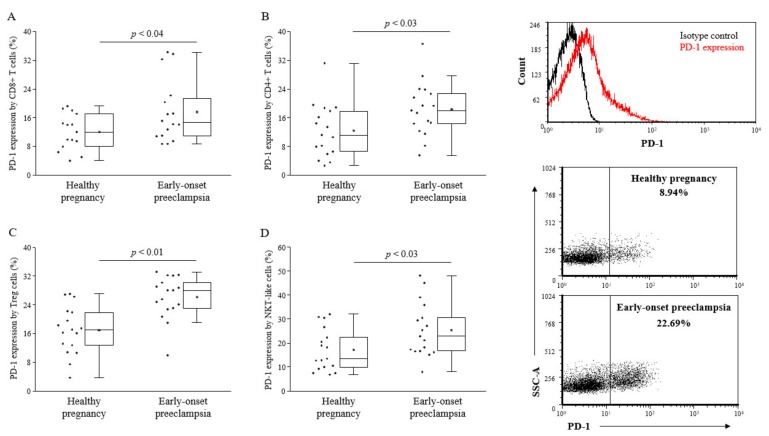
Programmed death-1 (PD-1) expression by peripheral lymphocyte populations in 3rd-trimester healthy pregnant women and in women with early-onset preeclampsia. The expression of PD-1 by CD8+ T cells (**A**), CD4+ T cells (**B**), Treg cells (**C**) and NKT-like cells (**D**) in peripheral blood of women with early-onset preeclampsia and in healthy pregnant women. The solid bars represent medians of 17 and 17 determinations, respectively, the boxes indicate the interquartile ranges, and the lines show the most extreme observations. Differences were considered statistically significant for *p*-values ≤ 0.05.

Analyzing the PD-1 expression by different lymphocyte subsets, CD8+ T cells, CD4+ T cells, and Tregs, NKT-like cells showed a significant upregulation of the PD-1 checkpoint molecule in the case of early-onset preeclampsia compared to healthy pregnant women (Figure 1). NK cells express a negligible amount of PD-1 molecule both in the 3rd-trimester of healthy pregnancy as well as in early-onset preeclampsia (data not shown).

### 2.3. PD-L1 Expression by Different Lymphocyte Subsets of the Innate and the Adaptive Immunity of 3rd-Trimester Healthy Pregnant Women and in Women with Early-Onset Preeclampsia

**Figure 2:**
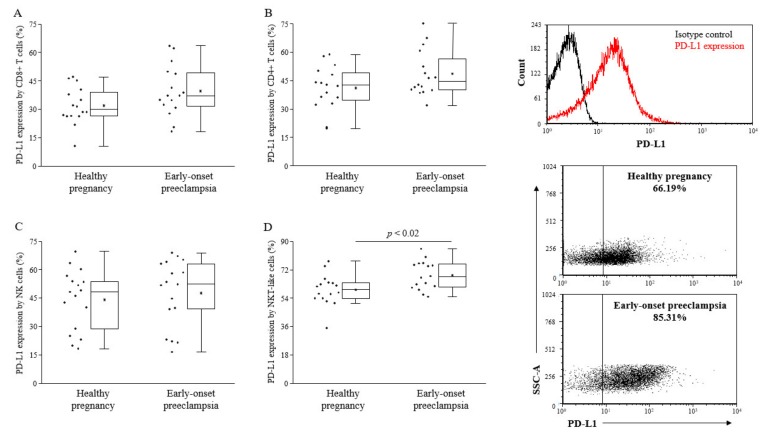
PD-L1 expression by peripheral lymphocyte populations in 3rd-trimester healthy pregnant women and in women with early-onset preeclampsia. The expression of PD-L1 CD8+ T cells (**A**), CD4+ T cells (**B**), NK cells (**C**), and NKT-like cells (**D**) in peripheral blood of women with early-onset preeclampsia and in healthy pregnant women. The solid bars represent medians of 17 and 17 determinations, respectively, the boxes indicate the interquartile ranges, and the lines show the most extreme observations. Differences were considered statistically significant for *p*-values ≤ 0.05.

Because of the broader expression profile of PD-L1 [23], we investigated the surface expression of the PD-L1 ligand on different lymphocyte subsets. We found significantly elevated levels of PD-L1 on NKT-like cells in early-onset preeclamptic patients compared to the healthy pregnant individuals in the 3rd-trimester (Figure 2).

### 2.4. Cytotoxicity of PD-1 Expressing Lymphocytes in the Peripheral Blood of 3rd-Trimester Healthy Pregnant Women and Women in with Early-Onset Preeclampsia

**Figure 3:**
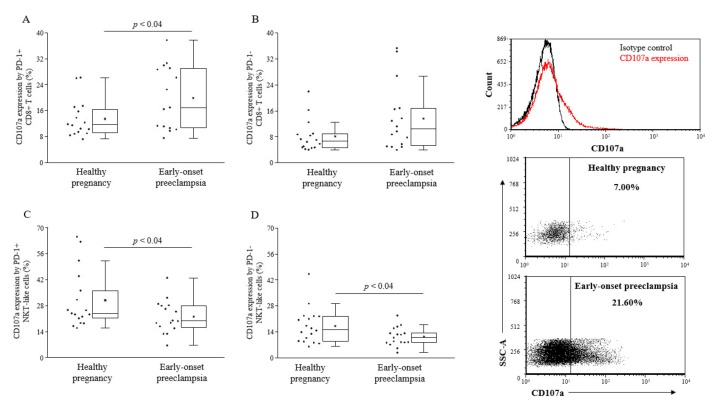
Cytotoxicity of peripheral CD8+ T cells and NKT-like cells in 3rd-trimester healthy pregnant women and in women with early-onset preeclampsia. The expression of CD107a by PD-1 positive and negative CD8+ T cells (**A** and **B**) and NKT-like cells (**C** and **D**) in peripheral blood of women with early-onset preeclampsia and in healthy pregnant women. The solid bars represent medians of 17 and 17 determinations, respectively, the boxes indicate the interquartile ranges, and the lines show the most extreme observations. Differences were considered statistically significant for *p*-values ≤ 0.05.

Cytotoxic activity of PD-1 or expressing lymphocytes was determined by the CD107a degranulation assay. While cytotoxicity of PD-1+ CD8+ T cells was found to be elevated in early-onset preeclampsia, both PD-1 positive and negative NKT-like cells showed reduced lytic activity compared to healthy pregnancy (Figure 3).

### 2.5. PD-1 and NKG2D Co-Expression by Lymphocytes in the Peripheral Blood of 3rd-Trimester Healthy Pregnant Women and in Women with Early-Onset Preeclampsia

**Figure 4:**
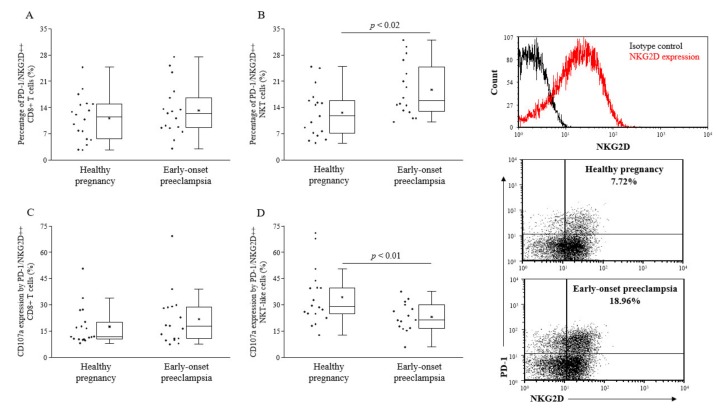
Percentage and cytotoxicity of PD-1/NKG2D double positive peripheral CD8+ T (**C**) and NKT-like cells (**D**) in 3rd-trimester healthy pregnant women and in women with early-onset preeclampsia. The percentage of PD-1/NKG2D double positive, CD8+ T cells (**A**), and NKT-like cells (**B**) in peripheral blood of women with early-onset preeclampsia and in healthy pregnant women. The solid bars represent medians of 17 and 17 determinations, respectively, the boxes indicate the interquartile ranges, and the lines show the most extreme observations. Differences were considered statistically significant for *p*-values ≤ 0.05.

Co-expression of PD-1 with the NK cell activating receptor NKG2D was determined on different lymphocyte subpopulations. The percentage of PD-1+ NKG2D+ NKT-like cells was found to be significantly increased in early-onset preeclamptic women compared to healthy 3rd-trimester pregnant women (Figure 4B). Regarding cytotoxicity, PD-1+ NKG2D+ NKT-like cells showed reduced cytotoxicity in the case of preeclampsia compared to healthy 3rd-trimester pregnancy (Figure 4D).

### 2.6. Serum-Soluble Programmed Death-Ligand 1 (sPD-L1) Levels in 3rd-Trimester Healthy Pregnant Women and in Women with Early-Onset Preeclampsia

**Figure 5:**
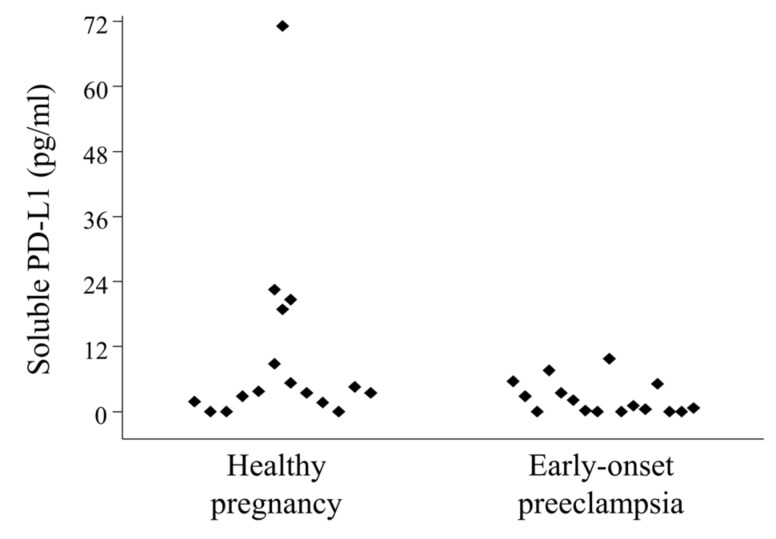
Circulating serum-soluble programmed death-ligand 1 (sPD-L1) levels in 3rd-trimester healthy pregnant women and in women with early-onset preeclampsia. Serum sPD-L1 levels in 16 healthy pregnant women and in 16 women with early-onset preeclampsia. Differences were considered statistically significant for *p*-values ≤ 0.05.

The median concentration of serum sPD-L1 in 3rd-trimester healthy pregnant women was 4.575 (range 1.668–71.136) pg/mL, significantly not different than in women with early-onset preeclampsia, which was 2.119 (range 0.217–9.759) pg/mL (Figure 5).

## 3. Discussion

Early-onset preeclampsia is thought to be a placental disorder [15]. Only theories exist about how it develops into a systemic maternal inflammatory response with an increased Th1/Th2 ratio. Current thinking focuses on the pro-inflammatory action of substances released by the decompensated and oxidatively stressed placenta [17].

However, the disturbed balance between immunotolerance and immunoactivation suggests the failure of immune regulation allowing exaggeration of inflammatory responses. Early-onset preeclampsia is characterized by a diminished Treg frequency, a well-known alteration suggesting generalized immune imbalance [24,25,26,27,28,29]. In this work, we could only detect a slight decrease in the number of Tregs in preeclamptic patients. In turn, decreased concentrations of Tregs affect and alter Th17/Treg ratio.

Beside Tregs, there are important elements at the molecular level involved in controlling immune responses. Immune checkpoint molecules play a critical role in limiting Th1 oriented immunity since they exert inhibitory effects upon TCR activation/antigen stimulation. In this work, we wondered whether the PD-1/PD-L1 pathway could be involved in the immunological imbalance in different lymphocytes subsets during the clinical phase of early-onset preeclampsia. In the case of CD4+ T cells, CD8+ T cells, and NKT-like cells, there is a significant upregulation of the PD-1 co-inhibitory receptor in patients with early-onset preeclampsia compared to healthy individuals, while its ligand PD-L1 is significantly elevated on the cell surface by NKT-like cells only. These findings indicate an enhanced presence of the PD-1/PD-L1 axis in early-onset preeclampsia, theoretically creating a more likely condition for interacting and damping inflammation as observed in healthy pregnancy. Yet, compared to healthy pregnant controls, cytotoxicity of PD1+ CD8+ T lymphocytes in early-onset preeclamptic women is further increased, while cytotoxicity of PD1 negative CD8+ T lymphocytes remains unchanged. This observation also gave us food for thought, since we have a situation here where upregulation of the PD-1/PD-L1 pathway is associated with the lack of its activity. Although the majority of NKT-like cells are conventional CD8+ T cells expressing NK associated receptors, the cytotoxic potential of these cells is significantly reduced in early-onset preeclampsia in contrast to healthy 3rd-trimester pregnancy, but there was no difference between the cytotoxicity of PD1+ and PD1- subpopulations in NKT-like cells. Further investigations are needed to clarify whether the failure of the PD-1/PD-L1 co-inhibitory pathway is partial in controlling inflammatory responses, affecting only cellular cytotoxicity. Fas ligand expression and probably cytokine production by CD8+ T cells and NKT-like cells could still be regulated by the PD-1 mediated pathway in early-onset preeclampsia.

On Tregs, while PD-1 expression was found to be increased in early-onset preeclampsia compared to healthy pregnancy, PD-L1 expression did not show any significant change (data not shown). In preeclampsia, if there is a small amount of Tregs without alteration of PD-L1 expression, peripheral induction of further Tregs from naive T cells through PD-L1 (Treg)/PD-1 (naive T cell) interaction could be diminished as well. These findings are in line with previous reports suggesting that the reduced number and enhanced PD-1 expression by Treg cells in early-onset preeclampsia are important factors with a major contribution to the missing immune control in this condition [30].

Recent studies have revealed that PD-L1 also has a soluble form [31,32]. This soluble form increases the complexity and diversity of the composition and function of the PD-1/PD-L1 signaling pathway. However, the exact role of this molecule remains unknown [33]. A couple of studies found that sPD-L1 is a negative therapeutic and prognostic biomarker in malignant tumors by facilitating immune-escape [34,35,36,37], while others could not confirm it [38]. In our hands, there is no difference between the sPD-L1 level in healthy pregnancy and in early-onset preeclamptic patients suggesting no role of the soluble ligand in the immunopathology of preeclampsia.

## 4. Materials and Methods

### 4.1. Patients

17 singleton pregnant women with the classic symptoms of preeclampsia (hypertension, proteinuria, and edema) were included in our study. Early-onset preeclampsia was defined by increased blood pressure (≥140 mmHg systolic or ≥90 mmHg diastolic on ≥2 separate occasions at least 4 h apart within a 24 h period) that occurred before the 34th week of gestation in a woman with previously normal blood pressure, accompanied by organ failure, such as significant proteinuria (≥0.3 g protein in 24-h urine collection in the absence of urinary tract infection). 17 healthy pregnant women appropriately matched for gestational age were recruited to form the control group (Table 2). None of the healthy women had any significant medical history, current or recent illnesses, or were taking medications.

### 4.2. Lymphocyte Separation, Cryopreservation and Thawing

Peripheral blood mononuclear cells (PBMC) were separated from heparinized venous blood on Ficoll-Paque (GE Healthcare, Little Chalfont, UK) gradient. Samples were washed in RPMI 1640 medium (Lonza, Basel, Switzerland) then counted and centrifuged. Cell pellet resuspension was performed in human serum containing 10% DMSO (Sigma-Aldrich, Budapest, Hungary) for cryoprotection. Cells were aliquoted in cryovials and stored in a −80 °C mechanical freezer. On the day of fluorescent cell labeling, cryovials were warmed up as quickly as possible in a 37 °C water bath and dimethyl sulfoxide (DMSO) was washed out twice in RPMI 1640 medium.

### 4.3. Antibodies

Thawed PBMC were used for surface and intracellular staining and analysis. The following monoclonal antibodies were used: Brilliant Violet (BV421)-conjugated anti-human PD-L1 (BD Biosciences, San Diego, CA, USA), BV510-conjugated anti-human CD3 (BD Biosciences, San Diego, CA, USA), fluorescein isothiocyanate (FITC)-conjugated anti-human CD4 (BD Biosciences, San Diego, CA, USA), FITC-conjugated anti-human CD107a (BD Biosciences, San Diego, CA, USA), phycoerythrin (PE)-conjugated anti-human PD-1 (Beckmann-Coulter, Miami, FL, USA), PE/Cy7-conjugated anti-human NKG2D (BD Biosciences, San Diego, CA, USA), allophycocyanin (APC)-conjugated anti-human CD56 (BD Biosciences, San Diego, CA, USA), APC-conjugated anti-human FoxP3 (eBioscience, San Diego, CA, USA), APC/H7-conjugated anti-human CD8 (BD Biosciences, San Diego, CA, USA).

### 4.4. Labeling of Lymphocytes and Flow Cytometric Analysis

**Figure 6:**
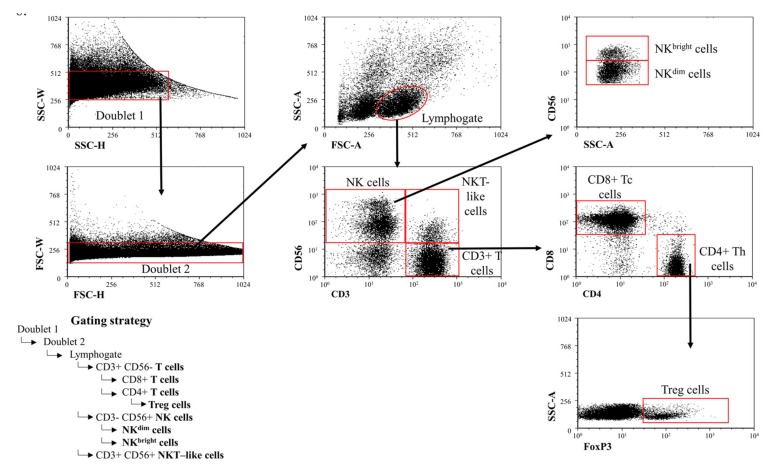
Gating strategy to detect peripheral immune cell populations. Gating technique used to detect immune cell populations in the periphery.

10^6^ thawed PBMC in a 100 µl PBS/tube was incubated for 30 min at room temperature with the fluorochrome-conjugated monoclonal antibodies. Immune cells were characterized (Figure 6) by surface staining using the antibodies shown in the previous chapter. Finally, the cells were resuspended in 300 µl PBS containing 1% paraformaldehyde (PFA) and stored at 4 °C in dark until FACS analysis. Labeled cells were measured with a BD FACSCanto II flow cytometer (BD Immunocytometry Systems, Erembodegem, Belgium) with the BD FACSDiva V6 software for data acquisition. Flow cytometric data analysis were performed with FCS express V4.

### 4.5. CD107a Functional Assay

The CD107a assay was set up based on a publication by Alter et al. [39]. Before surface labeling, PBMC were incubated for 4 h at 37 °C in the presence of FITC-conjugated anti-human CD107a monoclonal antibody in RPMI 1640 medium containing 10% fetal bovine serum, penicillin and streptomycin, ionomycin (Sigma-Aldrich, Budapest, Hungary), and phorbol myristate acetate (Sigma-Aldrich, Budapest, Hungary). Cells were washed and resuspended in PBS then stained with antibodies to CD8+ T and NKT-like cell markers together with PE-conjugated anti-human PD-1 antibody for 30 min at room temperature at dark. The cells were washed in PBS, fixed with 1% PFA and evaluated by FACS.

### 4.6. FoxP3 Intracellular Labeling

To define the phenotype of Treg cells, intracellular staining of Foxp3 was performed using the FoxP3 Staining Buffer Set (eBioscience, San Diego, CA, USA) according to the manufacturer’s protocol. Briefly, cells were permeabilized in 1 mL fixation/permeabilization buffer (Concentrate/Diluent 1:4) at 4 °C for 1 h, washed twice in the buffer, and then stained with the anti-human FoxP3 monoclonal antibody at 4 °C for 1 h. The cells were washed twice in the buffer, fixed with 1% PFA, and evaluated by FACS.

### 4.7. Determination of Serum Levels of Soluble PD-L1

Serum samples were collected from 3rd-trimester healthy pregnant women and from women with early-onset preeclampsia. 10 mL venous blood was taken to a disposable sterile test tube and centrifuged at 2000 rpm for 10 min. Serum samples were then transferred in 1 mL aliquots into cryovials and stored frozen at −80 °C until analysis. Serum PD-L1 concentration was measured by a Human PD-L1 DuoSet ELISA in 16 healthy pregnant women and in 16 women with early-onset preeclampsia according to the manufacturer’s protocol (R&D Systems, Minneapolis, MN, USA). Antibody duosets contained streptavidin- and biotin-labeled capture and detection antibodies, as well as appropriate standard material for PD-L1.

### 4.8. Statistical Analysis

Statistical analysis was performed using statistical software SPSS (IBM New York, NY, USA) version 23 package. Statistical comparisons were made using Student’s *t*-test. Differences were considered significant if the *p* value was equal to or less than 0.05.

### 4.9. Ethical Approval

The study was approved (02 April 2016) by the Regional Ethics Committee at the Medical School, University of Pecs (approved protocol registration number: 6149), and written informed consent was obtained from all patient. The study protocol conforms to the ethical guidelines of the 1975 Declaration of Helsinki.

## 5. Conclusions

Taken together, enhanced expression of either PD-1 or its ligand PD-L1, or both, on the cell surface of effector cells (T cells, NK cells, NKT-like cells) and Tregs suggests the possible involvement of the PD-1/PD-L1 pathway in the pathogenesis of the inflammatory stage of early-onset preeclampsia. According to our results, we hypothesize that PD-1 expression does not correlate with effector cells exhaustion, rather it may be some type of actual cell activation marker and it is associated with the failure of the axis to downregulate Th1 responses, contributing thereby to the exaggerated immunoactivation observed in early-onset preeclampsia.

## Figures and Tables

**Table 1:** Peripheral blood mononuclear cell phenotype characteristics in women with early-onset pre-eclampsia and in healthy pregnant women.

Lymphocyte Subpopulations	Healthy Pregnant Women (*n* = 17)	Early-Onset Preeclamptic Patients (*n* = 17)	*p*-Value
CD3+ T cells	68.80 ± 9.91	65.01 ± 11.53	NS
CD4+ T cells	38.15 ± 10.77	35.76 ± 9.94	NS
CD4+ T cells in CD3+ T cells	60.12 ± 8.66	55.57 ± 9.04	NS
CD8+ T cells	26.36 ± 13.16	23.17 ± 6.79	NS
CD8+ T cells in CD3+ T cells	32.66 ± 8.60	34.46 ± 7.78	NS
Treg cells	1.95 ± 1.30	1.73 ± 0,53	NS
NK cells	12.08 ± 5.52	15.62 ± 9.16	NS
NK^dim^ cells	10.37 ± 5.56	14.08 ± 8.59	NS
NK^bright^ cells	1.79 ± 0.65	1.60 ± 1.12	NS
NKT cells	2.91 ± 2.79	2.80 ± 1.62	NS

Statistical comparisons were made using the Student’s *t*-tests. The results were expressed as the mean value ± standard deviation of the mean (SD). Differences were considered significant when the value of *p* was equal to or less than 0.05. NS: not significant.

**Table 2:** Patients’ demographic and gynecological characteristics.

Parameter	Healthy Pregnant Women	Early-Onset Preeclamptic Patients
No. of patients	17	17
Age (years)	32.29 (26–43)	28.00 (17–42)
Gestational age at birth (week)	39.14 ± 1.10	32.35 ± 3.46 *
Gestational age at sampling (week)	33.29 ± 3.96	30.76 ± 2.41
Birth weight (gram)	3444.29 ± 512.38	1631.76 ± 738.56 *

The results were expressed as the mean value ± standard deviation of the mean (SD). * *p* < 0.05 vs. healthy pregnant women.

## Abbreviations

MAIT cell	Mucosal associated invariant T cell
NK cell	Natural Killer cell
NKT-like cell	Natural Killer T-like cell
PD-1	Programmed death-1 receptor
PD-L1	Programmed death-ligand 1
sPD-L1	Soluble programmed death-ligand 1
Treg cell	Regulatory T cell
